# Long-Term Exposure to 6-PPD Quinone Inhibits Glutamate Synthesis and Glutamate Receptor Function Associated with Its Toxicity Induction in *Caenorhabditis elegans*

**DOI:** 10.3390/toxics13060434

**Published:** 2025-05-26

**Authors:** Wei Wang, Yunhui Li, Dayong Wang

**Affiliations:** 1 Key Laboratory of Environmental Medicine Engineering of Ministry of Education, School of Public Health, Southeast University, Nanjing 210009, China; 2 Medical School, Southeast University, Nanjing 210009, China

**Keywords:** 6-PPDQ toxicity, glutamate metabolism, glutamate receptor, nematode

## Abstract

6-PPD quinone (6-PPDQ) is widely distributed in environments. In *Caenorhabditis elegans*, we first examined the effects of 6-PPDQ on glutamate synthesis and receptor function by analyzing glutamate content, related gene expression, and phenotypes after RNAi of these genes. Moreover, we performed glutamate treatment after 6-PPDQ exposure to determine the potential pharmacological effects of glutamate against 6-PPDQ toxicity. After exposure, the glutamate content was reduced by 0.1–10 μg/L 6-PPDQ, which was due to decreased expression of *W07E1.1*, *glna-1/2/3*, and *alh-6* governing glutamate synthesis from α-ketoglutarate, glutamine, and proline. RNAi of *W07E1.1*, *glna-1/2/3*, and *alh-6* decreased glutamate content in 6-PPDQ-exposed nematodes, and caused susceptibility to 6-PPDQ toxicity. Among glutamate transporter genes, *glt-1* expression was decreased by 0.1–10 μg/L 6-PPDQ. Moreover, 0.1–10 μg/L 6-PPDQ decreased glutamate receptor genes (*glr-1*, *glr-2*, and *glr-4*), and their expression was decreased by RNAi of *W07E1.1*, *glna-1/2/3*, *alh-6*, and *glt-1*. RNAi of these receptor genes resulted in susceptibility to 6-PPDQ toxicity, and *daf-7*, *jnk-1*, and *dbl-1* were identified as targets of neuronal *glr-1*, *glr-2*, and *glr-4*. Furthermore, 5 mM glutamate suppressed 6-PPDQ toxicity and increased expression of *glr-1*, *glr-2*, and *glr-4*. Our results demonstrated the risk of 6-PPDQ exposure in disrupting glutamate synthesis and affecting function of glutamate receptors, which was related to 6-PPDQ toxicity induction.

## 1. Introduction

N-(1,3-dimethylbutyl)-N’-phenyl-p-phenylenediamine (6-PPD), an antioxidant against oxidizing agents, has been widely utilized in tires [1]. 6-PPD can be transformed to 6-PPDQ after its reaction with ozone [2]. For 6-PPDQ, its widespread environmental occurrence was observed, especially in an aquatic environment [3]. In aquatic environments, 6-PPDQ concentrations range from ng/L to tens of μg/L [4]. Initially, because of the induction of acute lethality, 6-PPDQ exposure risk received much attention [5]. After that, some other aspects of 6-PPDQ toxicity were found in environmental animals, such as neurotoxicity and intestinal damage [6,7]. In mammals, damage to the liver and lung [8,9], and toxicity on sperm quality and neuronal mitochondria were also induced by 6-PPDQ [10,11]. More importantly, it was detected in human cerebrospinal fluid, urine, and blood [12,13], suggesting the health risks after 6-PPDQ exposure.

High sensitivity to pollutant exposure has been frequently observed in *Caenorhabditis elegans* [14,15,16,17]. Especially, after exposure to environmentally relevant concentrations (ERCs) of pollutants, their toxicity could be detected in nematodes [18,19,20]. In this animal model, intestinal oxidative stress, inhibited reproductive capacity, and suppressed locomotion were caused by 6-PPDQ at ERCs [21,22]. Exposure to 6-PPDQ also reduced lifespan [23] and induced immunosenescence [24]. Additionally, 6-PPDQ induced mitochondrial dysfunction associated with toxicity against mitochondrial complexes [25,26]. Due to conserved metabolic processes and regulation mechanisms, *C. elegans* is useful for determining the molecular basis of biochemical metabolism [27]. Moreover, the metabolism of lipids, glucose, and glycogen were disrupted by 6-PPDQ at ERCs [28,29,30], and an alteration in glucose metabolism was even observed in offspring after 6-PPDQ exposure [31].

**Figure 1:**
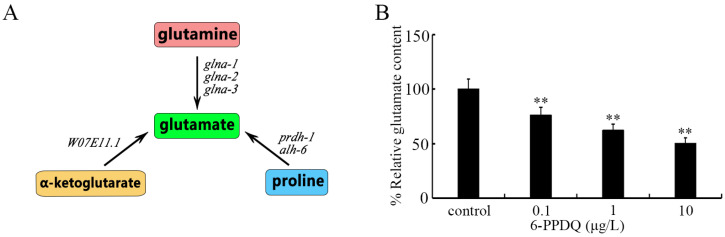
Effects of 6-PPDQ exposure on glutamate content. (**A**) Diagram showing biochemical basis for synthesis of glutamate in nematodes. (**B**) Effect of 6-PPDQ exposure on glutamate content. ** *p* < 0.01 vs. control.

Glutamate is not only an important neurotransmitter [32] but also a metabolite [33]. It was reported that glutamate treatment could inhibit both the uptake and toxicity of cadmium [34], which suggests a beneficial role of glutamate against pollutant toxicity. In cells, glutamate synthesis has three biochemical sources, including α-ketoglutarate, glutamine, and proline (Figure 1A). In nematodes, W07E11.1 is a glutamate synthase governing glutamate synthesis from α-ketoglutarate (https://wormbase.org), *GLNA*-1/2/3 are glutaminases governing glutamate synthesis from glutamine [35], PRDH-1 and ALH-6 are proline dehydrogenases (PRODHs) (https://wormbase.org), and 1-pyrroline-5-carboxylate dehydrogenase (P5CDH) [36] governs glutamate synthesis from proline. *EAT*-4 and *GLT*-1/3/4/5/6/7 are glutamate transporters [37,38,39]. *GLR*-1/2/3/4/5/6/7/8 are glutamate receptors [40]. Thus, we first investigated the possible effect of 6-PPDQ on glutamate synthesis and the function of glutamate receptors in nematodes. Moreover, an association of this effect with 6-PPDQ toxicity was examined. Our observations suggest a disruption in glutamate synthesis and the function of glutamate receptors by 6-PPDQ at ERCs, which was further related to 6-PPDQ toxicity induction.

## 2. Materials and Methods

### 2.1. Animal Maintenance

Nematodes were grown on NGM plates fed by *E. coli* OP50 [41]. Adult hermaphroditic *C. elegans* nematodes were treated with bleaching solution (10% NaClO and 0.45 M NaOH) to collect embryos [42], which would develop to synchronize populations at L1 larval stage.

### 2.2. 6-PPDQ Exposure

6-PPDQ was procured from Toronto Research Chemicals Co. (Vaughan, ON, Canada), with a purity >97%. 6-PPDQ exposure concentrations were established at 0.1–10 μg/L based on reported ERCs of 6-PPDQ [4]. L1 larvae were exposed to 6-PPDQ for 6.5 days [21]. During exposure, 6-PPDQ solutions were refreshed daily. To fulfill nutritional need of larvae, OP50 was supplemented within 6-PPDQ solutions.

### 2.3. Glutamate Content

Glutamate content was measured using glutamate quantification kit from Sangon Biotech. Co. (Shanghai, China). For glutamate content assay, approximately 0.1 g nematodes were homogenized in ice bath. After homogenization and centrifugation, supernatants were added with reagents in the kit. Mixtures were incubated for 30 min for colorimetric reaction and absorbance measurement at 340 nm. Experiments were repeated three times.

### 2.4. Gene Expression

Animals were collected in tubes and lysed using pre-cooled TRIzol at 4 °C. cDNA was prepared using M-MuLV reverse transcriptase. Quantitative real-time polymerase chain reaction (qRT-PCR) analysis was performed utilizing SYBR Green RT-qPCR master mix (Vazyme, Nanjing, China). *tba-1* was reference gene [43]. Three replicates were carried out. Primers are shown in Table S1.

### 2.5. RNA Interference (RNAi)

Double-stranded RNA-expressing *E. coli* HT115 was prepared to feed L1 larval nematodes [44]. HT115 expressing L4440 (empty vector) was used as the control [45]. TU3401 is a transgenic strain for neuronal RNAi of genes. The efficiency of the RNAi was evaluated using qRT-PCR, and the results are depicted in Figure S1.

### 2.6. Endpoints

For assay of reactive oxygen species (ROS), nematodes were incubated in 1 mM CM-H_2_DCFDA for 3 h [22]. Intestinal fluorescence signals were observed using fluorescence microscope under FITC channel. Images were analyzed after normalization with autofluorescence. Fifty nematodes were analyzed per group. Head thrash and body bend were chosen to assess change of locomotion behavior. The frequency of head swings in one minute was recorded as head thrash, and number of times that nematodes’ body bent as it completed the movement in 20 s was classified as body bend [46]. Fifty nematodes were analyzed per group. Experiments were repeated three times.

### 2.7. Exogenous Glutamate Treatment

After 6-PPDQ (10 μg/L) exposure, nematodes were treated with 5 mM glutamate for 24 h [47]. Experiments were carried out in triplicate.

### 2.8. Data Analysis

Statistical tests were carried out using SPSS v27. Difference among groups was evaluated using one-way or two-way ANOVA (for multi-factor comparison) followed by post hoc test. *p*-value of <0.01 (**) was deemed statistically significant.

## 3. Results

### 3.1. 6-PPDQ Decreased Glutamate Content

After 6-PPDQ exposure, the glutamate content was significantly decreased (Figure 1B). Additionally, after 6-PPDQ exposure, this decrease in glutamate content was concentration-dependent (Figure 1B).

### 3.2. 6-PPDQ Decreased Expression of Genes Governing Glutamate Synthesis

**Figure 2:**
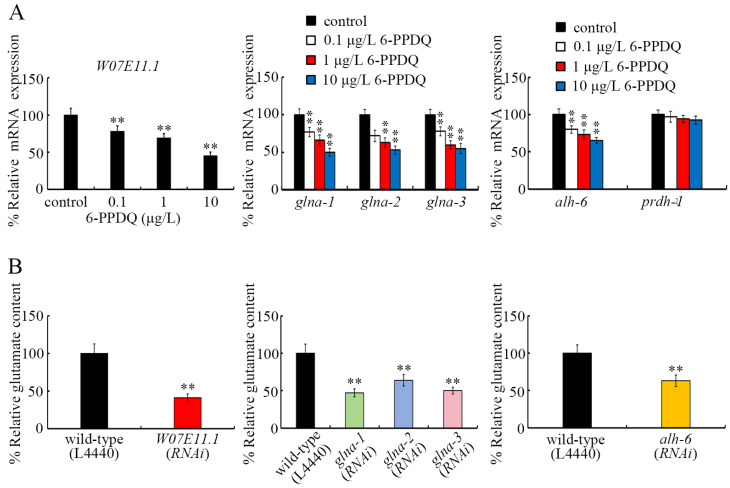
Effect of 6-PPDQ exposure on glutamate synthesis. (**A**) Effect of 6-PPDQ exposure on expression of genes governing glutamate synthesis. ** *p* < 0.01 vs. control. (**B**) Effect of RNAi of *W07E11.1*, *glna-1*, *glna-2*, *glna-3*, and *alh-6* on glutamate content in 6-PPDQ-exposed nematodes. Exposure concentration of 6-PPDQ was 10 μg/L. ** *p* < 0.01.

The expression of *W07E11.1*, which governs glutamate synthesis from α-ketoglutarate, was decreased by 0.1–10 μg/L 6-PPDQ (Figure 2A). Meanwhile, glutamate content was inhibited by *W07E11.1* RNAi (Figure 2B).

The expression of *glna-1*, *glna-2*, and *glna-3*, which govern glutamate synthesis from glutamine, was decreased by 0.1–10 μg/L 6-PPDQ (Figure 2A). After 6-PPDQ exposure, RNAi of these three genes reduced glutamate content (Figure 2B).

The *alh-6* expression was decreased by 0.1–10 μg/L 6-PPDQ, but 6-PPDQ exposure did not alter *prdh-1* expression (Figure 2A). After 6-PPDQ exposure, *alh-6* RNAi also suppressed glutamate content (Figure 2B).

### 3.3. RNAI of W07E11.1, glna-1, glna-2, glna-3, and alh-6 Induced Susceptibility to 6-PPDQ Toxicity

**Figure 3:**
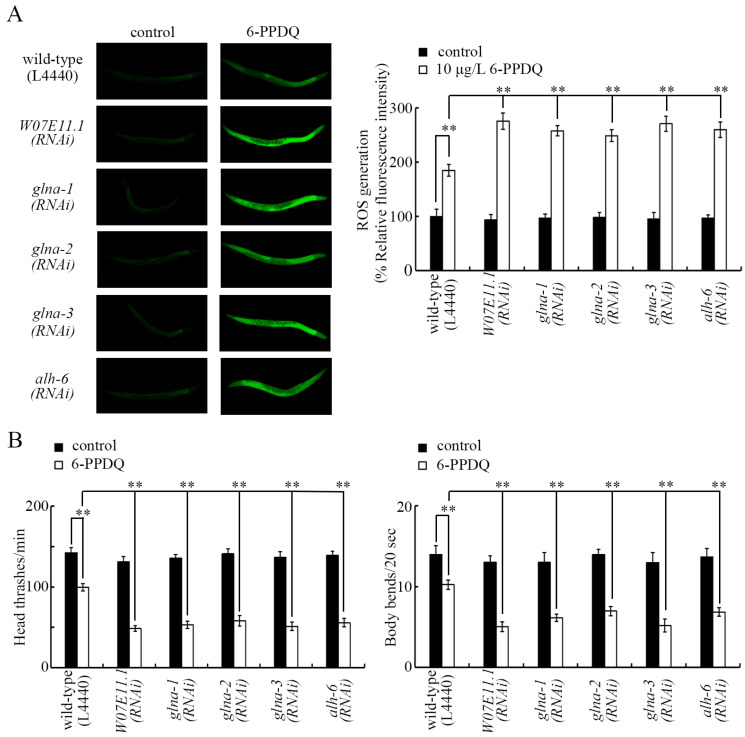
Effect of RNAi of *W07E11.1*, *glna-1*, *glna-2*, *glna-3*, and *alh-6* on 6-PPDQ toxicity in causing ROS generation (**A**) and decrease in locomotion (**B**). Exposure concentration of 6-PPDQ was 10 μg/L. ** *p* < 0.01.

We next investigated the association of an alteration in genes governing glutamate synthesis with 6-PPDQ toxicity induction. ROS generation and locomotion were used as endpoints. In 6-PPDQ-exposed nematodes, both ROS generation and decrease in locomotion were strengthened by *W07E11.1*, *glna-1*, *glna-2*, *glna-3*, and *alh-6* RNAi (Figure 3A,B), suggesting susceptibility to 6-PPDQ in nematodes with RNAi of these genes.

### 3.4. 6-PPDQ Decreased Expression of Glutamate Transporter Gene glt-1

**Figure 4:**
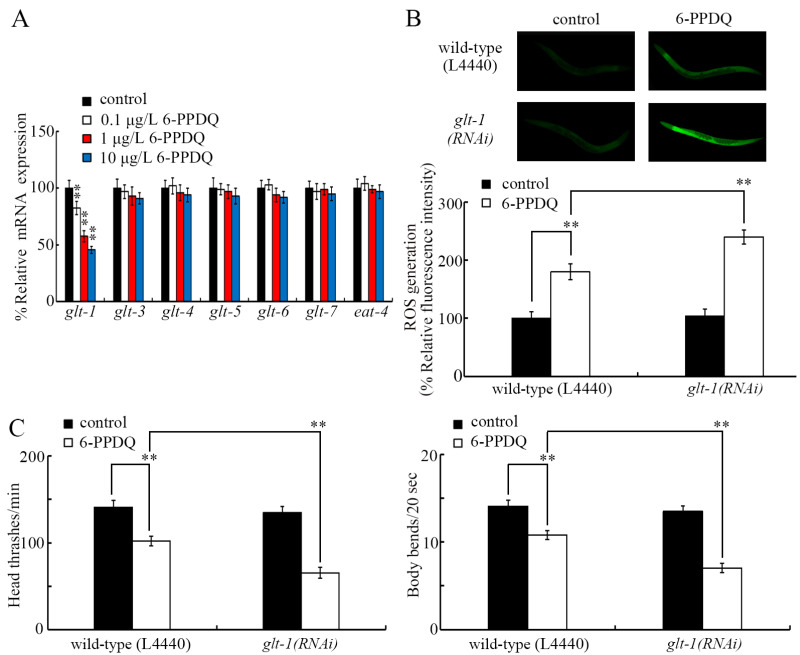
Effect of 6-PPDQ exposure on expression of glutamate transporter genes. (**A**) Effect of 6-PPDQ exposure on expression of *eat-4*, *glt-1*, *glt-3*, *glt-4*, *glt-5*, *glt-6*, and *glt-7*. ** *p* < 0.01 vs. control. (**B**) Effect of RNAi of *glt-1* on 6-PPDQ toxicity in causing ROS generation. Exposure concentration of 6-PPDQ was 10 μg/L. ** *p* < 0.01. (**C**) Effect of RNAi of *glt-1* on 6-PPDQ toxicity in decreasing locomotion. Exposure concentration of 6-PPDQ was 10 μg/L. ** *p* < 0.01.

Among the glutamate transporter genes, *glt-1* expression was significantly decreased by 0.1–10 μg/L 6-PPDQ, but the expression of *eat-4* and *glt-3-7* was not changed by 0.1–10 μg/L 6-PPDQ (Figure 4A). RNAi of *glt-1* resulted in susceptibility to 6-PPDQ toxicity (Figure 4B,C).

### 3.5. 6-PPDQ Decreased Expression of the Glutamate Receptor Genes glr-1, glr-2, and glr-4

**Figure 5:**
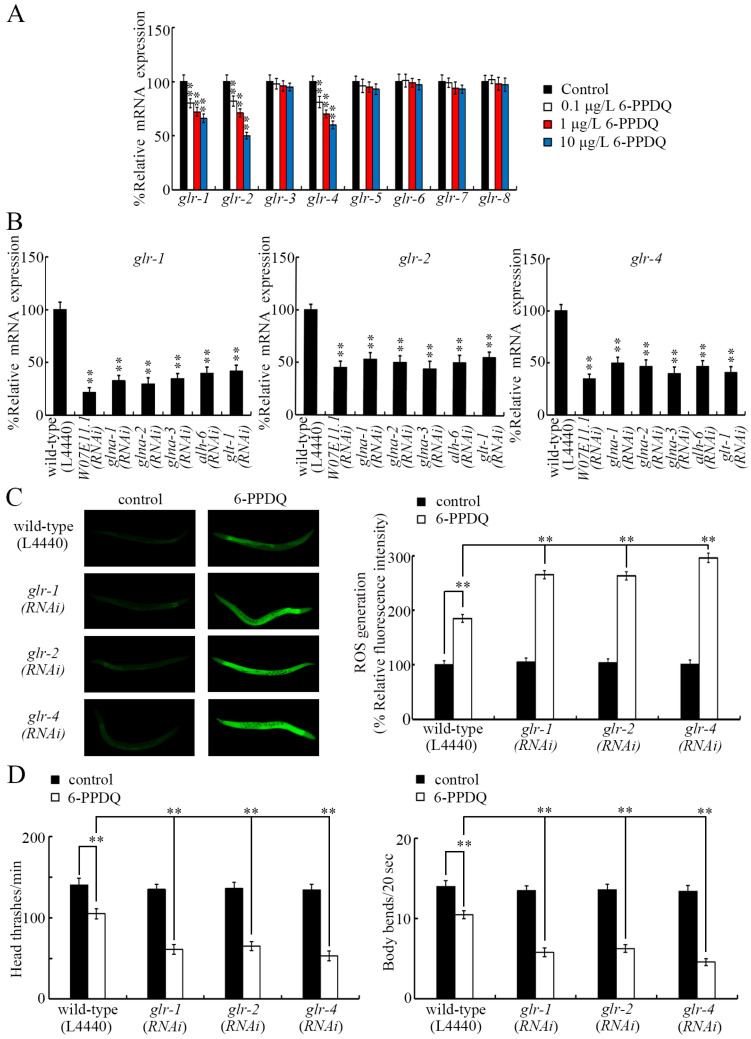
Effect of 6-PPDQ exposure on expression of glutamate receptor genes. (**A**) Effect of 6-PPDQ exposure on expression of *glr-1*, *glr-2*, *glr-3*, *glr-4*, *glr-5*, *glr-6*, *glr-7*, and *glr-8*. ** *p* < 0.01 vs. control. (**B**) Effect of RNAi of *W07E11.1*, *glna-1*, *glna-2*, *glna-3*, *alh-6*, and *glt-1* on expression of *glr-1*, *glr-2*, and *glr-4* in 6-PPDQ-exposed nematodes. Exposure concentration of 6-PPDQ was 10 μg/L. ** *p* < 0.01 vs. wild-type (L4440). (**C**) Effect of RNAi of *glr-1*, *glr-2*, and *glr-4* on 6-PPDQ toxicity in causing ROS generation. Exposure concentration of 6-PPDQ was 10 μg/L. ** *p* < 0.01. (**D**) Effect of RNAi of *glr-1*, *glr-2*, and *glr-4* on 6-PPDQ toxicity in decreasing locomotion. Exposure concentration of 6-PPDQ was 10 μg/L. ** *p* < 0.01.

Among the glutamate receptor genes, the expression of *glr-1*, *glr-2*, and *glr-4* was inhibited by 0.1–10 μg/L 6-PPDQ, but the expression of *glr-3*, *glr-5*, *glr-6*, *glr-7*, and *glr-8* was not altered by 0.1–10 μg/L 6-PPDQ (Figure 5A). After 6-PPDQ exposure, the expression of *glr-1*, *glr-2*, and *glr-4* was suppressed by RNAi of genes governing glutamate synthesis (*W07E11.1*, *glna-1/2/3*, and *alh-6*), as well as RNAi of *glt-1* (Figure 5B).

The ROS generation induced by 6-PPDQ was enhanced by *glr-1*, *glr-2*, and *glr-4* RNAi (Figure 5C). Similarly, 6-PPDQ-induced locomotion inhibition was strengthened by *glr-1*, *glr-2*, and *glr-4* RNAi (Figure 5D).

### 3.6. GLR-1, GLR-2, and GLR-4 Regulated 6-PPDQ Toxicity by Affecting DAF-7, JNK-1, and DBL-1

**Figure 6:**
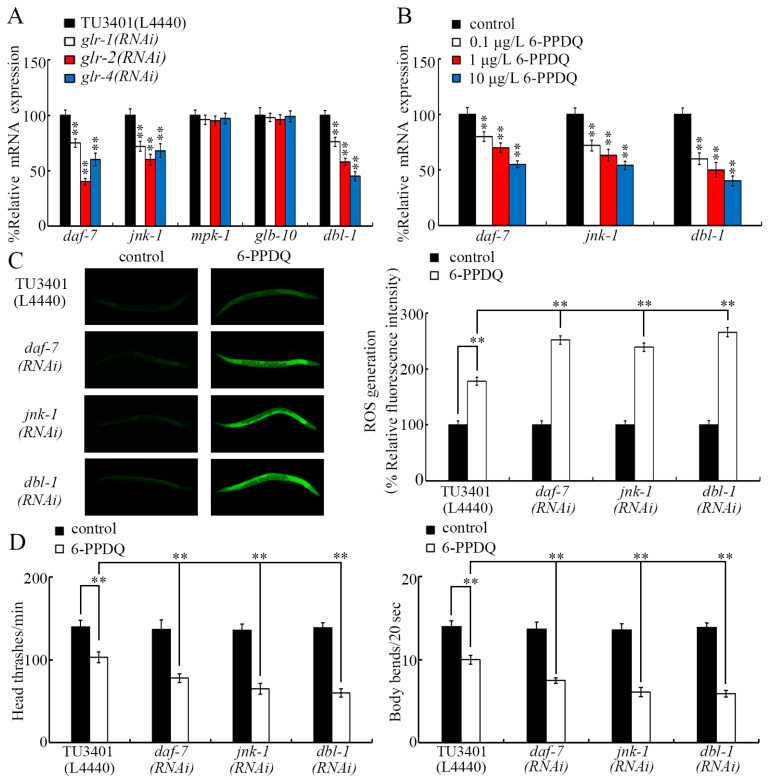
Neuronal RNAi of *glr-1*, *glr-2*, and *glr-4* affected expression of *daf-7*, *jnk-1*, and *dbl-1* in 6-PPDQ-exposed nematodes. (**A**) Effect of neuronal RNAi of *glr-1*, *glr-2*, and *glr-4* on expression of *daf-7*, *jnk-1*, *mpk-1*, *glb-10*, and *dbl-1* in 6-PPDQ-exposed nematodes. Exposure concentration of 6-PPDQ was 10 μg/L. ** *p* < 0.01 vs. TU3401 (L4440). (**B**) Effect of 6-PPDQ exposure on expression of *daf-7*, *jnk-1*, and *dbl-1*. ** *p* < 0.01 vs. control. (**C**) Effect of neuronal RNAi of *daf-7*, *jnk-1*, and *dbl-1* on 6-PPDQ toxicity in causing ROS generation. Exposure concentration of 6-PPDQ was 10 μg/L. ** *p* < 0.01. (**D**) Effect of neuronal RNAi of *daf-7*, *jnk-1*, and *dbl-1* on 6-PPDQ toxicity in decreasing locomotion. Exposure concentration of 6-PPDQ was 10 μg/L. ** *p* < 0.01.

*GLR*-1, *GLR*-2, and *GLR*-4 are predominantly expressed in neurons (https://wormbase.org). Regarding pollutant toxicity, some molecular signals (*DAF*-7, *JNK*-1, *MPK*-1, *GLB*-10, and *DBL*-1) act in the neurons to carry out their functions [48]. After 6-PPDQ exposure, the expression of *daf-7*, *jnk-1*, and *dbl-1* was suppressed by neuronal RNAi of *glr-1*, *glr-2*, and *glr-4*, but the expression of *mpk-1* and *glb-10* was not changed by neuronal RNAi of *glr-1*, *glr-2*, and *glr-4* (Figure 6A). Meanwhile, 6-PPDQ (0.1–10 μg/L) decreased *daf-7*, *jnk-1*, and *dbl-1* expression, and this decrease in their expression was concentration-dependent (Figure 6B). Moreover, the ROS generation and inhibition of locomotion induced by 6-PPDQ were strengthened by neuronal RNAi of *daf-7*, *jnk-1*, and *dbl-1* (Figure 6C,D).

### 3.7. Pharmacological Effect of Glutamate Treatment on 6-PPDQ Toxicity

**Figure 7:**
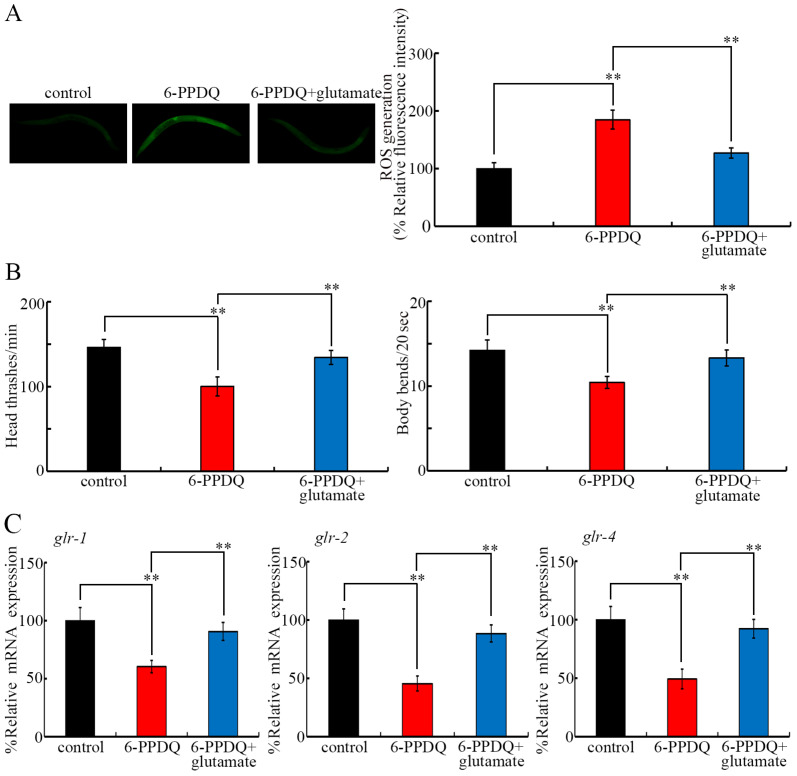
Effect of glutamate (5 mM) treatment on toxicity induced by 6-PPDQ (10 μg/L). (**A**) Effect of glutamate treatment on ROS generation in 6-PPDQ-exposed nematodes. (**B**) Effect of glutamate treatment on locomotion in 6-PPDQ-exposed nematodes. (**C**) Effect of glutamate treatment on expression of *glr-1*, *glr-2*, and *glr-4* in 6-PPDQ-exposed nematodes. ** *p* < 0.01.

ROS generation induced by 10 μg/L 6-PPDQ was inhibited following 5 mM glutamate treatment (Figure 7A). Similarly, the decrease in locomotion induced by 10 μg/L 6-PPDQ was suppressed by treatment with 5 mM glutamate (Figure 7B). Moreover, the decrease in *glr-1*, *glr-2*, and *glr-4* expression in 6-PPDQ-exposed nematodes was further inhibited by treatment with 5 mM glutamate (Figure 7C).

## 4. Discussion

*C. elegans* can be used to determine mechanisms of biochemical metabolism, such as glucose and lipid metabolism [27,49]. 6-PPDQ caused an accumulation of lipids, glucose, and glycogen in *C. elegans* [28,29,50]. Additionally, dopamine metabolism in *C. elegans* has also been shown to be disrupted by 6-PPDQ [51]. Glutamate content was further reduced by 0.1–10 μg/L 6-PPDQ (Figure 1B). Thus, 6-PPDQ at ERCs potentially disrupts the biochemical metabolism of multiple compounds.

In nematodes, W07E11.1 is predicted to govern glutamate synthesis from α-ketoglutarate, and its expression was decreased by 6-PPDQ (Figure 2A). GLNA-1/2/3 govern glutamate synthesis from glutamine [35], and their expression was also inhibited by 6-PPDQ (Figure 2A). PRDH-1 and ALH-6 are required for glutamate synthesis from proline [36], and only *alh-6* expression was suppressed by 6-PPDQ (Figure 2A). After 6-PPDQ exposure, glutamate content was reduced by *W07E1.1*, *glna-1/2/3*, and *alh-6* RNAi (Figure 2B), which confirmed the functions of these genes in controlling glutamate synthesis. Therefore, the decrease in expression of *W07E1.1*, *glna-1/2/3*, and *alh-6* provided an important biochemical basis for the reduction in glutamate content induced by 6-PPDQ exposure.

Susceptibility to 6-PPDQ toxicity could be caused by RNAi of *W07E1.1*, *glna-1/2/3*, and *alh-6* (Figure 3). This observation suggests that a decrease in the expression of genes of enzymes governing glutamate synthesis from α-ketoglutarate, glutamine, and proline contributes to 6-PPDQ toxicity induction. A mutation in *alh-6* could cause premature reproductive senescence [52], and ALH-6 could be considered a biomarker of age-related muscle changes [53].

Among the glutamate transporter genes, only *glt-1* expression showed sensitivity to 6-PPDQ exposure. The *glt-1* expression was decreased by 0.1–10 μg/L 6-PPDQ (Figure 4A). Meanwhile, susceptibility to 6-PPDQ toxicity in causing ROS generation and locomotion inhibition was caused by *glt-1* RNAi (Figure 4B,C). Similar to the function of the identified genes governing glutamate synthesis, this suggests that the inhibition of GLT-1 can also mediate 6-PPDQ toxicity.

Among eight glutamate receptor genes, three (*glr-1*, *glr-2*, and *glr-4*) were found to be involved in the control of 6-PPDQ toxicity. RNAi of these three glutamate receptor genes caused susceptibility to 6-PPDQ toxicity (Figure 5C,D). Meanwhile, the expression of these three glutamate receptor genes was inhibited by 6-PPDQ (Figure 5A). These observations suggest that the inhibition of these three glutamate receptors mediates the toxicity induction of 6-PPDQ. The expression of the other five glutamate receptor genes was not altered by 6-PPDQ (Figure 5A), implying that the expression of these receptor genes may not be sensitive to 6-PPDQ exposure. RNAi of *glr-4* can cause susceptibility to nanoplastic toxicity [54]. The behavioral response of *C. elegans* to Al_2_O_3_-NPs exposure has been shown to be mediated by GLR-2 [40]. In nematodes, *glr-1* expression has also been shown to be decreased by lindane exposure [55]. Moreover, after 6-PPDQ exposure, we found that the expression of *glr-1*, *glr-2*, and *glr-4* was decreased by RNAi of *W07E11.1*, *glna-1/2/3*, *alh-6*, and *glt-1* (Figure 5B), suggesting that a decrease in the expression of genes governing glutamate synthesis and transport can cause the further inhibition of receptors after 6-PPDQ exposure. These receptors are predominantly expressed in neurons (https://wormbase.org). In addition to neurons, W07E11.1, GLNA-1/2/3, ALH-6, and GLT-1 are also expressed in the intestine, gonads, muscle, and/or epidermis (https://wormbase.org), which suggests that the glutamate is synthesized and further transported from different tissues to the neuron to activate the GLR-1, GLR-2, and GLR-4 receptors, thereby exerting its effects.

Considering that these glutamate receptors are predominantly expressed in the neurons of *C. elegans*, we identified targets of these three glutamate receptors in the neurons to control 6-PPDQ toxicity. DAF-7, JNK-1, and DBL-1 were identified as targets for neuronal *GLR*-1, *GLR*-2, and *GLR*-4 in controlling 6-PPDQ toxicity. Three aspects of evidence were raised to support this. Firstly, the expression of *daf-7*, *jnk-1*, and *dbl-1* was decreased by neuronal RNAi of *glr-1*, *glr-2*, and *glr-4* in 6-PPDQ-exposed nematodes (Figure 6A). Secondly, the expression of *daf-7*, *jnk-1*, and *dbl-1* was decreased by 6-PPDQ (Figure 6B). Thirdly, neuronal RNAi of *daf-7*, *jnk-1*, and *dbl-1* caused susceptibility to 6-PPDQ (Figure 6C,D). DAF-7 and DBL-1 are two TGF-β ligands [56,57]. JNK-1 is a JNK MAPK [54]. RNAi of *daf-7*, *jnk-1*, and *dbl-1* has also been shown to result in susceptibility to the toxicity of other pollutants, such as nanoplastics and CDDP quinone [48,58,59]. In nematodes, it remains unclear whether GLR-1, GLR-2, and GLR-4 regulate 6-PPDQ toxicity by activating or inhibiting other downstream targets; thus, this needs further determination.

In the current study, ROS generation and locomotion inhibition, which are two aspects of toxicity induced by 6-PPDQ, were suppressed following glutamate treatment (Figure 7A,B). Similarly, glutamate treatment inhibited the toxicity of heavy metals such as cadmium [30]. These observations suggest that glutamate treatment can potentially be used as a pharmacological intervention against damage caused by 6-PPDQ. Nevertheless, more studies are needed to further confirm this interventional strategy in mammals. Moreover, the 6-PPDQ-induced inhibition of the expression of glutamate receptor genes was suppressed following glutamate treatment (Figure 7C). This further supports the role of glutamate in inhibiting 6-PPDQ toxicity by activating these glutamate receptor genes. After 6-PPDQ exposure, glutamate synthesis was suppressed, and the resulting reduction in glutamate content may lead to the inhibition of glutamate receptors, which, in turn, causes susceptibility to 6-PPDQ toxicity.

## 5. Conclusions

In conclusion, the reduction in glutamate content by 6-PPDQ was observed in nematodes, which was due to the inhibition of expression of genes governing glutamate synthesis from α-ketoglutarate, glutamine, and proline. The expression of glutamate transporter gene *glt-1* and receptor genes (*glr-1*, *glr-2*, and *glr-4*) was also decreased by 6-PPDQ, and susceptibility to 6-PPDQ toxicity was observed in nematodes after RNAi of genes governing glutamate synthesis, transporter gene *glt-1*, and receptor genes. Some molecular signals (DAF-7, JNK-1, and DBL-1) were identified as downstream targets of neuronal glutamate receptors (GLR-1, GLR-2, and GLR-4) to control 6-PPDQ toxicity. Pharmacological treatment with glutamate further supported the role of glutamate in modulating 6-PPDQ toxicity by affecting corresponding receptor genes. Nevertheless, considering the simple developmental structure of *C. elegans*, additional studies in mammals still need to be carried out.

## Data Availability

The original data presented in this study are included in the article/Supplementary Materials; further inquiries can be directed to the corresponding author.

## Supplementary Materials

The following supporting information can be downloaded at: https://www.mdpi.com/article/10.3390/toxics13060434/s1. Figure S1: RNAi efficiency of *W07E11.1*, *glna-1*, *glna-2*, *glna-3*, *glt-1*, *glr-1*, *glr-2*, and *glr-4*. ** *p* < 0.01 vs. wild-type (L4440); Table S1. Primer information for qRT-PCR.
