# Supplementary material for: Long-Term Exposure to 6-PPD Quinone Inhibits Glutamate Synthesis and Glutamate Receptor Function Associated with Its Toxicity Induction in Caenorhabditis elegans
Source: Toxics. 2025 May 26;13(6):434. doi: 10.3390/toxics13060434 (PMC12197550; doi:10.3390/toxics13060434)
Supplement: Supplementary file 1 [file toxics-13-00434-s001.zip › toxics-3621622-supplementary.pdf]

Supporting Information:

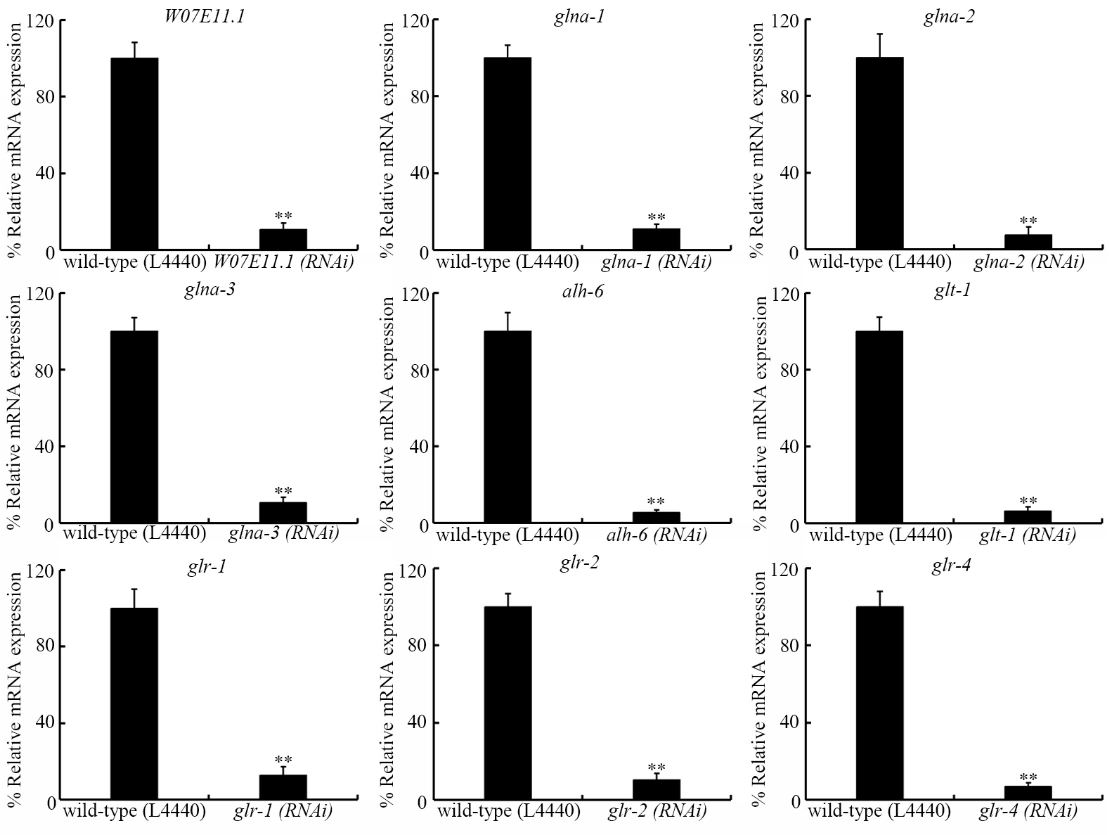

Fig. S1 RNAi efficiency of *W07E11.1, glna-1, glna-2, glna-3, glt-1, glr-1, glr-2,* and *glr-4*. $^{**}P < 0.01$ *vs* wild-type (L4440).

Table S1. Primer information for qRT-PCR

Gene	Forward primer (5'-3')	Reverse primer (5'-3')
W07E11.1	GGGCAAGACAACATTGGCAG	TCCAGCTCCTTCAGTCCACA
glna-1	CAGCTCCCGCAAAACATTCC	CAGGGAGCCGGGTAACAAATA
glna-2	CAGAATTTGCAGACGTGTTCG	TGCTCTTTGGTGAGTTTCCA
glna-3	TGGTGCCATTGTAGTCGCTT	AATGTTGCAGCCATCACAGC
alh-6	ATCCGTCTTTCTGTCGGCTG	ACATCGCGAGCTCTATAAAGGA
prdh-1	TGTGTCCGTAATGGTGGCAA	TCTAGCGTTTCGGCAGTTTT
glt-1	CACAAAACATGCCGCCAGAT	ACCAGTAACAACAGCCACGG
glt-3	AATCTGTCGTGCTCGGTGTT	TGCTGGAATGTTGCCTGGAT
glt-4	TGCATCTCCGGATCCAAAAC	TCCCCTAAAAAGTGTATTAGCCAC
glt-5	CAAGGCAATGGTTCTGCCAC	TTTGCGAGTGCTTTGGCTTC
glt-6	TCTAGGGGATGCGTTTGCTG	TCTCGCGGAAAGAGGAGAGA
glt-7	AAGCGATGAGCAGTGTGGAA	GGGCGATCAATCTCGGCTAA
eat-4	TACGGAGCTGCTGAGACTAC	TCGCAACTAGGCAATAGAACTT
glr-1	GTGGAACATGGTGAACGCAG	AGTGATGGTAGGTGGAGGCT
glr-2	TCGACTGGGAGCCTTCAAAA	AGGGCTCTCAAAAATGCACG
glr-3	GGTGAATCCCGTCTTGGAGT	AGTAGGGGGTGACAGGTGAA
glr-4	TGGAAAATGAGTAAGTTCAACTCG	TGTTCATGTGTTGCTGCTGG
glr-5	GTGGTGAAGCTGAGATGGCA	ACCAAAATCACATCCGCCCT
glr-6	GAGTTCTTTGTGCGGTGCTG	ACCAAGGCCATCCTGCTATG
glr-7	AGGAAAGCAAGAAAAGCCGC	AGTGCAAGAAATCCCGTTGG
glr-8	GGATTTGAAACGTGCCCAGG	CGTTTGGAAAGCCACATGGT
daf-7	GAGCAGGATATGGGCGTTGA	GGTCTGTTGGGGATGAAGGG
dbl-1	ACAAACAGGCAACAGCGAAC	TCAGTAGGCACACAGCAAGG
glb-10	CTGGCACAAATCGGCTACAC	CTCCTTTAGTTCCGCCAAGC
jnk-1	CGTATCCGTCACATCCAGGTAG	ATCCAAAGAGACAGCGTCGT
mpk-1	AACCACGAAACATGATGCCAAC	CTCCGTCGGCCATCTTTCTAT
tba-1	TCAACACTGCCATCGCCGCC	TCCAAGCGAGACCAGGCTTCAG
